# A rare case of uniparental disomy 9 concomitant with low-level mosaicism for trisomy 9

**DOI:** 10.18699/vjgb-25-68

**Published:** 2025-09

**Authors:** A.S. Iakovleva, Zh.G. Markova, L.A. Bessonova, N.V. Shilova

**Affiliations:** Bochkov Research Centre for Medical Genetics, Moscow, RussiaBochkov Research Centre for Medical Genetics, Moscow, Russia; Bochkov Research Centre for Medical Genetics, Moscow, Russia; Bochkov Research Centre for Medical Genetics, Moscow, Russia; Bochkov Research Centre for Medical Genetics, Moscow, Russia

**Keywords:** mosaicism, trisomy 9, uniparental disomy 9, сhromosomal microarray analysis (CMA), FISH, мозаицизм, трисомия хромосомы 9, однородительская дисомия хромосомы 9, хромосомный микроматричный анализ (ХМА), FISH

## Abstract

Uniparental disomy of chromosome 9, in combination with low-level mosaicism for chromosome 9, represents a rare chromosomal disorder. One of the mechanisms underlying the formation of uniparental disomy is the trisomy rescue, which concurrently results in low-level mosaicism. The diagnosis of mosaic aneuploidies poses significant challenges due to the limited sensitivity and resolution of conventional cytogenetic methods, which often fail to detect low-level mosaicism. Additionally, the variable distribution of cell lines within the patient’s tissues, as well as the heterogeneity of samples derived from the same tissue, complicates the precise determination of the impact of mosaic trisomy on the phenotypic expression. Phenotypic manifestations associated with mosaic trisomy 9 are characterized by considerable variability. During the prenatal period, intrauterine growth restriction is frequently observed in cases of this chromosomal abnormality, although this finding is not pathognomonic for the condition. In liveborn infants with trisomy 9 mosaicism, characteristic phenotypic features may include craniofacial anomalies (such as micrognathia and ear malformations), scoliosis, low-set ears, feeding and respiratory difficulties, hip dysplasia, seizures, and developmental delays. To establish a diagnosis in a patient presenting with multiple dysembryogenic stigmata and psychomotor retardation, a comprehensive molecular cytogenetic analysis was conducted. This included high-resolution chromosomal microarray analysis (CMA) and fluorescence in situ hybridization (FISH) using targeted DNA probes. CMA identified regions of loss of heterozygosity (LOH) on chromosome 9, indicative of uniparental disomy, and suggested the presence of low-level mosaicism for trisomy 9. Subsequent FISH analysis of cultured lymphocytes, employing DNA probes specific to various regions of chromosome 9, confirmed the low-level mosaicism for trisomy 9. The results of our study are consistent with the idea that mosaicism for chromosome 9, particularly when combined with uniparental disomy, constitutes a complex genetic anomaly that can lead to a spectrum of phenotypic manifestations, including developmental delay, growth abnormalities, and behavioral anomalies. CMA and FISH are highly effective methods for the diagnosis of uniparental disomy and low-level mosaicism involving chromosome 9.

## Abstract (ru)

Однородительская дисомия хромосомы 9 в сочетании с мозаицизмом низкого уровня хромосомы 9 – редкое хромосомное нарушение. Один из механизмов формирования однородительской дисомии – коррекция анеуплоидии, приводящая также к мозаицизму низкого уровня. Трудности диагностики мозаичных анеуплоидий связаны с ограничениями в чувствительности и разрешающей способности стандартных цитогенетических методов, затрудняющих выявление мозаицизма низкого уровня. Различное соотношение клеточных линий в тканях пациента или неоднородность образцов одной и той же ткани не позволяют однозначно определить влияние мозаичной трисомии на формирование фенотипа пациента. Фенотипические признаки мозаичной трисомии 9 (Т9) отличаются широкой вариабельностью. В пренатальном периоде при этой хромосомной патологии часто отмечается внутриутробная задержка развития. У живорожденных с мозаицизмом по Т9 могут наблюдаться характерные фенотипические особенности, такие как черепно-лицевые аномалии (микрогнатия, аномалии ушей), сколиоз, низко посаженные уши, дисплазия тазобедренного сустава, судороги и задержка развития, а также проблемы с кормлением и дыханием. С целью установления диагноза у пациента с множествен ными стигмами дисэмбриогенеза и задержкой психомоторного развития проведено комплексное исследова- ние молекулярно-цитогенетическими методами, включающее хромосомный микроматричный анализ (ХМА) высокого разрешения и флуоресцентную гибридизацию in situ (FISH) с таргетными ДНК-зондами. ХМА показал наличие участков потери гетерозиготности на хромосоме 9, свидетельствующее об однородительской дисомии, и позволил предположить мозаицизм низкого уровня по Т9. Дополнительный FISH-анализ культивированных лимфоцитов с ДНК-зондами на различные районы хромосомы 9 позволил установить мозаицизм низкого уровня по Т9. Результаты нашего исследования согласуются с представлением о том, что мозаицизм по хромосоме 9 в сочетании с однородительской дисомией является сложной генетической аномалией, которая может привести к задержке развития, нарушению роста и особенностям поведения. ХМА и FISH – это эффективные методы диагностики однородительской дисомии и мозаицизма низкого уровня по трисомии хромосомы 9.

## Introduction

Uniparental disomy (UPD) is a genetic anomaly in which both homologs of a chromosome or chromosomal segment are inherited exclusively from one parent in contrast to normal meiotic segregation, wherein a child inherits one homolog from the father and one from the mother. The term UPD was first introduced in 1980 (Engel, 1980) based on the observation that a partial diploid DNA sequence, and occasionally an entire pair of chromosomes may be inherited solely from one parent. The clinical significance of this phenomenon was not recognized until the 1990s when UPD was established as one of the possible causes of two neurodevelopmental syndromes – Prader–Willi and Angelman syndromes (Cassidy, Schwartz, 1998). UPD is subdivided into isodisomy where two identi- cal copies of a chromosome are inherited from one parent, and heterodisomy where a pair of non-identical homologous chromosomes is inherited from one parent (Chen Q. et al., 2023). The frequency of UPD varies considerably among chromosomes (Eggermann et al., 2015). The highest UPD frequencies have been reported for acrocentric chromosomes 13, 14, 15, 21, and 22 due to their involvement in Robertsonian translocations – chromoso- mal rearrangements resulting from the fusion of the long arms and centromeres of two acrocentric chromosomes with the concomitant loss of material from their short arms

For most chromosomes, UPD has no clinical consequences. However, chromosomes 6, 7, 8, 11, 13, 14, 15, and 20 contain genes subject to parent-of-origin-specific expression (imprinting). UPD involving these chromosomes may lead to the development of corresponding imprinting disorders as well as autosomal recessive diseases or X-linked recessive disorders in females. Rare inheritance of both sex chromosomes from a single parent may underlie transmission of X-linked disorders from father to son (Del Gaudio et al., 2020).

The clinical manifestations of UPD may range from varying degrees of intellectual disability and/or syndromes involving multiple congenital anomalies to an asymptomatic presentation. However, UPD involving autosomes is most frequently associated with intrauterine growth restriction, dysmorphic features, or multiple congenital malformations (Kotzot, 2002)

The estimated prevalence of UPD is approximately one case per 5,000 live births (Liehr, 2010). An analysis of data from more than 4 million individuals tested by the private genetics company 23andMe, and 431,094 participants from the Northern European UK Biobank, demonstrated that UPD affecting any chromosome (not only those harboring imprinted regions) occurs at a frequency of one in 2,000 births. Given that the 23andMe dataset primarily includes healthy individuals from the general population, this figure is considered a more representative estimate of the population-level frequency of UPD. Data from 23andMe further suggest that UPD of chromosomes lacking imprinted genes or genes associated with autosomal recessive disorders is often not associated with a pathological phenotype (Del Gaudio et al., 2020).

The most common mechanism leading to UPD is chromosomal nondisjunction during meiosis or mitosis. The principal mechanisms of UPD formation include monosomy rescue, trisomy rescue, mitotic error, and gamete complementation (Nakka et al., 2019). Aneuploidy correction occurs either through loss of the third chromosome (trisomy rescue) or duplication of a monosomic chromosome (monosomy rescue). Trisomy rescue may occur as a result of anaphase lag and can contribute to UPD formation.

Trisomy 9 (T9) is a rare chromosomal abnormality that can occur in either mosaic or non-mosaic forms (Cantú et al., 1996). The regular (non-mosaic) form of T9 is incompatible with live birth and is identified in 2.2–2.7 % of spontaneous abortions occurring in the first trimester of pregnancy (Benn, Grati, 2021). Nevertheless, postnatally diagnosed patients with mosaic T9 have been reported (Bruns, Campbell, 2015). In most individuals with mosaic T9, prenatal fin- dings include intrauterine growth restriction or low fetal weight, oligohydramnios, placental insufficiency, premature rupture of membranes, and skeletal anomalies (Bruns, Campbell, 2015).

Postnatally mosaic T9 is typically characterized by multiorgan involvement, including craniofacial anomalies, malformations of the heart, genitourinary system, skeleton, and central nervous system, as well as abnormal ear morphology, micrognathia, and hip dysplasia. Most reported patients also experience prenatal and perinatal complications related to respiration, growth, and feeding (Li M. et al., 2021). The severity and frequency of developmental anomalies and intellectual disability correlate with the proportion of trisomic cells in various tissues (Lee et al., 2018).

Herein we present a rare case of chromosome 9 loss of heterozygosity in combination with low-level mosaicism for T9 identified in a patient with psychomotor developmental delay and congenital anomalies, using a molecular cytogenetic approach.

## Clinical description of the patient

The proband is a girl, 3 years and 7 months old at the time of examination, presenting with psychomotor developmental delay and feeding difficulties (does not chew solid food, experiences choking episodes). Parental ages at the time of birth were 37 years (mother) and 29 years (father). The child was born from the third pregnancy (I – miscarriage, II – maternal half-sister, 13 years old). Intrauterine growth restriction was diagnosed at 30 weeks of gestation. She was born with a birth weight of 2,657 g (10th percentile), length of 50 cm (10th percentile), head circumference of 32 cm (3rd percentile), and chest circumference of 31 cm (3rd percentile). Multiple minor anomalies (dysmorphic stigmata) of embryonic development were noted. Phenotype at the time of examination included: positional cranial deformation, enophthalmos, microtia, dysplastic auricular morphology, bilateral preauricular fistulas, congenital ptosis of the right upper eyelid, muscle hypotonia, bilateral mixed conductive hearing loss, and congenital dislocation of the right hip. Height: 89 cm (50th percentile), weight: 9.8 kg (<3rd percentile), head circumference: 45 cm (<3rd percentile).

Early psychomotor development: did not hold her head up, rolled from back to side by age one, does not sit independently; vocalizations present, reaches for objects and grasps them, makes spontaneous leg movements.

Karyotype: 46,XX – normal female.

Family history is negative for hereditary disorders.

Clinical exome sequencing (covering 6,640 genes), previously performed, revealed only variants of uncertain clinical significance (VUS).

## Materials and methods

Genomic DNA was extracted from a peripheral venous blood sample collected in EDTA using the Gentra Puregene Blood Kit Plus (Qiagen, California) according to the manufacturer’s protocol.Chromosomal microarray analysis (CMA) was performed using the high-density CytoScan® HD Array Kit in accordance with the manufacturer’s instructions (Affymetrix Inc., California, USA). Data were processed, analyzed, and normalized using Affymetrix Chromosome Analysis Suite (ChAS) version 4.0 with reference genome build NA33.1 (hg19).

Fluorescence in situ hybridization (FISH) was carried out according to the manufacturer’s protocols on chromosome preparations obtained from 72-hour peripheral blood lymphocyte cultures. DNA probes targeting chromosome 9 were used: pericentromeric heterochromatin of chromosome 9 (SE 9 classical), and subtelomeric regions of the short and long arms of chromosome 9 (Sub Telomere 9pter, Sub Telomere 9qter) (KREATECH, Netherlands). The analysis was performed using an AxioImager M.1 epifluorescence microscope (Carl Zeiss) and the Isis digital image analysis software (MetaSystems).

## Results

**Fig. 1.**
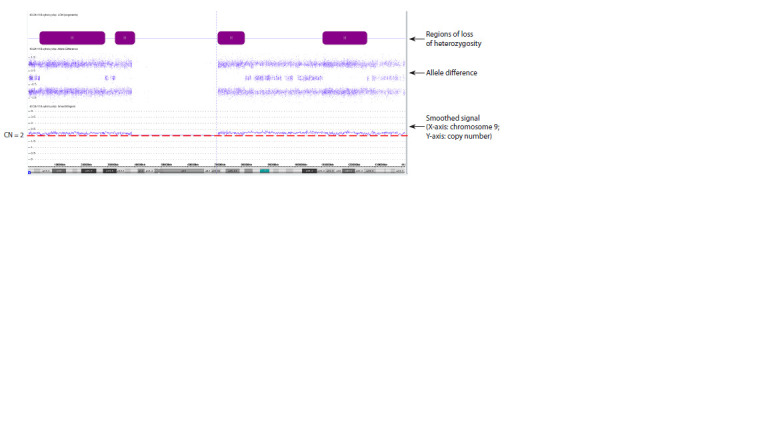
CMA results. Molecular karyotype of the patient (according to ISCN 2020): arr[GRCh37] 9p24.2p21.1(4284369_28756447)×2 hmz, 9p21.1p13.1(32561829_40087758)×2 hmz, 9q21.11q21.31(71013800_81233686)×2 hmz, 9q31.2q33.3(110291122_126976363)×2 hmz.

Chromosomal microarray analysis using high-density arrays with genotype-informative SNP probes revealed extensive regions of loss of heterozygosity (LOH) on chromosome 9. In parallel, the smooth signal indicated a slight shift toward increased copy number along the entire chromosome 9, suggesting the presence of low-level mosaicism for T9. The estimated level of T9 mosaicism ranged from 22 to 26 % in specific regions of the long arm of chromosome 9 (Fig. 1).

**Fig. 2.**
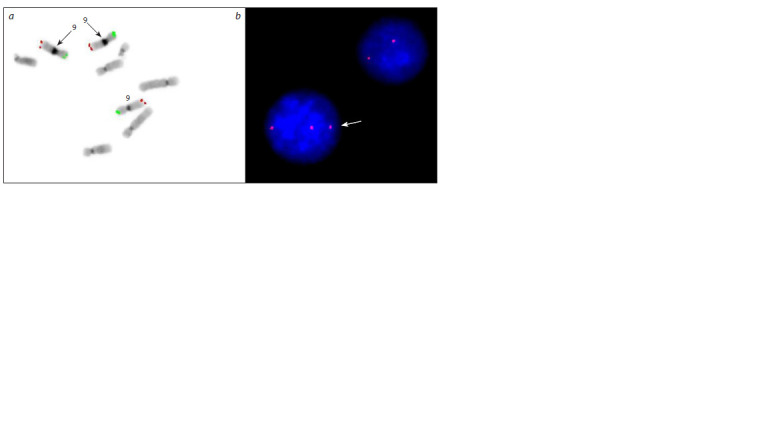
FISH analysis with DNA probes for chromosome 9. a – a metaphase spread hybridized with DNA probes targeting the subtelomeric regions of the short (green signal) and long (red signal) arms of chromosome 9 (inverted DAPI staining). Arrows indicate the chromosome 9 homologs each exhibiting identical pericentromeric heterochromatin block sizes; b – interphase nucleus hybridized with a DNA probe for the pericentromeric heterochromatin of the long arm of chromosome 9. The arrow indicates a nucleus with three hybridization signals corresponding to three copies of the 9q11.1 region.

Additional FISH analysis using DNA probes targeting the subtelomeric regions of the short and long arms of chromosome 9 confirmed the presence of a clone with trisomy involving the entire chromosome 9 (Fig. 2a). T9 was detected in six out of 100 metaphase spreads analyzed: ish 9(RH65569+, SHGC-149365+)×3[6/100]/9(RH65569+, SHGC-149365+)×2[94/100].

To assess the level of mosaicism, interphase FISH was performed using a DNA probe specific to the pericentromeric heterochromatin of the long arm of chromosome 9. Among 300 interphase nuclei analyzed, mosaicism with two distinct cell clones was identified: 6.7 % of cells exhibited three copies of the D9Z5 locus, and 93.3 % of cells contained two copies (Fig. 2b). FISH result: nuc ish(D9Z5×3)[20/300] (ISCN 2020).Analysis of metaphase spreads revealed a polymorphism in the pericentromeric heterochromatin region of chromosome 9. In disomic cells, both homologs exhibited heterochromatin blocks of identical size. This heterochromatin block polymorphism supports the presence of uniparental disomy (UPD) for chromosome 9 (Liehr, 2010). Thus, molecular cytogenetic analysis confirmed low-level mosaicism for T9. It can be hypothesized that following the correction of T9 in an initially aneuploid embryo, two cell clones emerged during ontogenesis – one trisomic and the other disomic with UPD of chromosome 9. Parental material was unavailable for analysis, and therefore the parental origin of the UPD could not be determined.

## Discussion

**Fig. 3.**
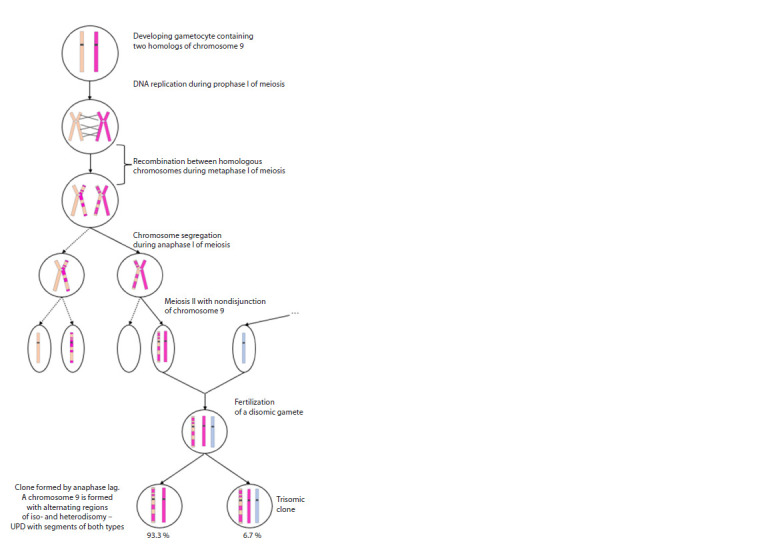
Schematic representation of the formation of mixed uniparental iso-/heterodisomy of chromosome 9 with T9 mosaicism

Chromosomal microarray analysis revealed four extended regions of homozygosity on chromosome 9, with a cumulative length of approximately 59 million base pairs, corresponding to 42 % of the total length of the chromosome. Based on the established theory that UPD can result from trisomy rescue, the observed LOH profile is more consistent with complete UPD of chromosome 9 than with segmental UPD. Since SNP array analysis is informative only for isodisomy, a mixed iso-/heterodisomy of chromosome 9 is likely (Fig. 3).

According to published molecular studies, extended regions of homozygosity on chromosome 9, detected by SNP arrays, most likely result from postzygotic trisomy rescue combined with mitotic recombination (Ma N. et al., 2023). This mechanism of UPD formation implies that low-level or cryptic mosaicism is likely present in many cases, particularly for chromosome 9 (Eggermann et al., 2018).

A particular diagnostic challenge in CPM involving trisomy is the risk of associated UPD, especially when the aneuploid chromosome harbors imprinted genes (Kotzot, 2002). Postzygotic correction of trisomy may occur in embryonic tissues, while the placenta remains partially or fully trisomic. The frequency of UPD in the setting of CPM is approximately 2 % (Malvestiti et al., 2015).

Current knowledge regarding the pathogenic mechanisms of UPD remains limited. UPD can affect phenotype through various pathways. The abnormal phenotype may result from imprinting, mosaicism for T9, or recessive mutations. In cases of isodisomy, the risk of monogenic disorders increases due to homozygosity for recessive alleles (Spence et al., 1988; Quan et al., 1997). For example, UPD of chromosome 9 has been associated with Leigh syndrome (Tiranti et al., 1999; Xiao et al., 2019), cartilage-hair hypoplasia (Sulisalo et al., 1997), and amyotrophic lateral sclerosis (Yang et al., 2014). Whole-exome sequencing in our patient did not reveal pathogenic variants on chromosome 9, suggesting the absence of autosomal recessive disorders

Another pathogenic effect of UPD may involve genomic imprinting which refers to parent-of-origin-specific gene expression. However, it has been shown that UPD of chromosome 9 does not result in an abnormal phenotype (Björck et al., 1999). Chromosome 9 harbors the imprinted gene GLIS3, which is expressed from the paternal allele, but this gene has not been linked to the clinical features observed in our patient.

Literature analysis shows that UPD of chromosome 9, whether maternal or paternal in origin, in the absence of trisomy, is not associated with developmental abnormalities or phenotypic deviations typically observed in T9 cases (see the Table)

**Table 1.**
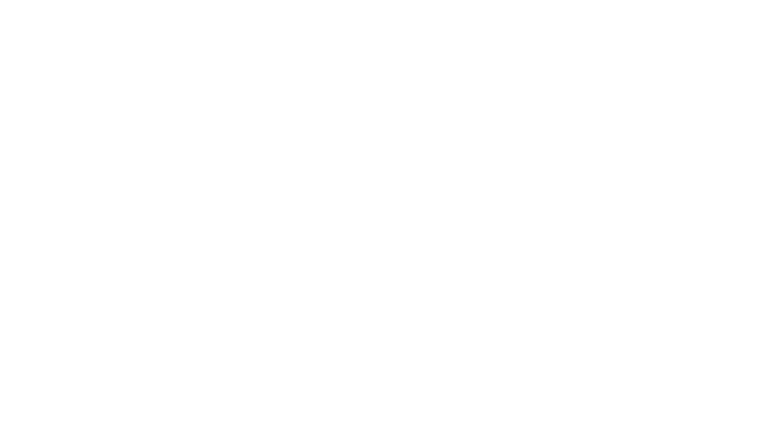
Published data on the correlation between clinical features, karyotype, and T9 Note. IUGR – intrauterine growth restriction; CP – cerebral palsy.

Patients with mosaic T9 exhibit a broad spectrum of clinical manifestations affecting multiple organ systems. Craniofacial dysmorphism, cardiac anomalies, genitourinary defects, skeletal and central nervous system abnormalities are most frequently observed. Global developmental delay is also commonly reported (Li M. et al., 2021). The mild phenotypic features observed in our patient are most likely attributable to the low proportion of T9-positive cells. Birth weight of 2,657 g (10th percentile), length of 50 cm (10th percentile), head circumference of 32 cm (3rd percentile), and chest circumference of 31 cm (3rd percentile) indicate intrauterine growth restriction, likely associated with placental dysfunction potentially caused by confined placental mosaicism for T9. Since no prenatal cytogenetic testing was conducted in this case, the presence of mosaicism for T9 in both extraembryonic and embryonic tissues can only be presumed. Nevertheless, several reported cases indicate favorable pregnancy outcomes in instances of UPD of chromosome 9 combined with low-level mosaic T9 diagnosed prenatally (Chen Q. et al., 2023).

FISH-based assessment of mosaicism significantly improves the sensitivity for detecting low-level mosaicism. Unfortunately, in our case, peripheral blood was the only available tissue. It is possible that FISH analysis of additional tissues – derived from different germ layers – could provide insights into the distribution of abnormal cells and their tissue-specific phenotypic effects

## Conclusion

UPD of chromosome 9 is a rare genetic anomaly, it may have clinical significance due to imprinting defects and the manifestation of autosomal recessive disorders

This clinical case describing a patient with UPD of chromosome 9 combined with low-level mosaicism for trisomy 9 (T9) diagnosed using chromosomal microarray analysis and fluorescence in situ hybridization demonstrates the effectiveness of combining modern molecular genetic techniques.

The coexistence of UPD and T9 mosaicism may lead to variable phenotypic manifestations depending on the degree of mosaicism and the distribution of the trisomic clone across different tissues (Ma N. et al., 2023).

To establish a correlation between UPD of chromosome 9 and an abnormal phenotype, it is necessary to analyze additional tissue samples accessible for examination (e. g., skin fibroblasts, buccal epithelium, or urinary sediment cells) in order to assess the extent and tissue distribution of mosaicism. The phenomenon of UPD requires further investigation with a focus on the identification of specific genetic abnormalities and mosaicism patterns, which may allow for more accurate prognostic assessment.

## Conflict of interest

The authors declare no conflict of interest.
